# Standard setting made easy: validating the Equal Z-score (EZ) method for setting cut-score for clinical examinations

**DOI:** 10.1186/s12909-020-02080-x

**Published:** 2020-05-25

**Authors:** Boaz Shulruf, Ying-Ying Yang, Pin-Hsiang Huang, Ling-Yu Yang, Chin-Chou Huang, Chia-Chang Huang, Chih-Wei Liu, Shiau-Shian Huang, Chen-Huan Chen, Fa-Yauh Lee, Shou-Yen Kao

**Affiliations:** 1 University of New South Wales, Sydney, Australia; 2 National Yang-Ming University, Taipei, Taiwan; 3 Taipei Veterans General Hospital, Taipei, Taiwan; 4 Taichung Veterans General Hospital, Yuli, Taiwan

**Keywords:** Borderline regression method, Equal Z-score method, Standard setting, Objective structural clinical examination

## Abstract

**Background:**

This study aims to assess the feasibility, reliability and validity of the panel-based Equal Z-score (EZ) method applied to objective structural clinical examination (OSCE) of Chinese medical students and undertaking a comparison with the statistical techniques-based Borderline Regression Method (BRM).

**Methods:**

Data received from two cohorts of 6th and 7th year medical students in Taiwan who set the mock OSCE as a formative assessment. Traditionally this medical school uses BRM to set the pass/fail cut-score. For the current study, 31 OSCE panellists volunteered to participate in the EZ method in parallel to the BRM.

**Results:**

In the conduct of this study, each panel completed this task for an OSCE exam comprising 12 stations within less than 60 min. Moreover, none of the 31 panellists, whose are busy clinicians, had indicated that the task was too difficult or too time-consuming. Although EZ method yielded higher cut-scores than the BRM it was found reliable. Intraclass correlation (ICC) measuring absolute agreement, across the three groups of panellists was .893 and .937 for the first and second rounds respectively, demonstrating high level of agreement across groups with the EZ method and the alignment between the BRM and the EZ method was visually observed. The paired t-test results identified smaller differences between the cut-scores within methods than across methods.

**Conclusions:**

Overall this study suggests that the EZ method is a feasible, reliable and valid standard setting method. The EZ method requires relatively little resources (takes about an hour to assess a 12 station OSCE); the calculation of the cut-score is simple and requires basic statistical skills; it is highly reliable even when only 10 panellists participate in the process; and its validity is supported by comparison to BRM. This study suggests that the EZ method is a feasible, reliable and valid standard setting method.

## Background

Commonly, standard setting methods aim to distinguish between competent and incompetent examinees who sit a test or an examination (these terms are used interchangeably). Standard setting was as an umbrella term, incorporating consensual approaches of panels of experts to set discrete cut-scores on continuous test performance scales [1]. Among the panel based standard setting methods, the most commonly used are the Angoff method [2, 3], the Hofstee and Beuk methods [4] and the Bookmark method [5]. In these methods, panellists who are experts in the assessed topic and familiar with the curriculum and the expected student level of performance, make judgment about item or the entire test difficulty. Then the examination’s cut-score is calculated from the aggregated panellists’ decisions.

There are also other standard setting methods which do not use expert panels but rather employ statistical techniques using test scores generated by examinees without further judgment. Among these are the Borderline Regression Method (BRM); The Objective Borderline Method (OBM), the Cohen Method [6–9]. It is however, commonly acceptable, that a panel based standard setting method, which is informed by examination results yielded from an advanced psychometric analysis such as Item Response Theory (IRT) [10] or Rasch models [11] (e.g. the Item Mapping and the Bookmark methods), provides trustworthy standards for educational examination [12, 13].

Recently, a few new standard setting methods have been introduced to the literature. Among them are the Cohen Method [14], The Objective Borderline Methods (OBM & OBM2) [15, 16] and a new method incorporating some principles form both the Angoff and the Hofstee methods [6], which has not yet been properly named [17]. The current study focuses on this new method [17] which after a consultation with the authors we decided to name it the Equal Z-score method, in short the ‘EZ method’, which may also be pronounced: the ‘easy method’.

**Fig. 1:**
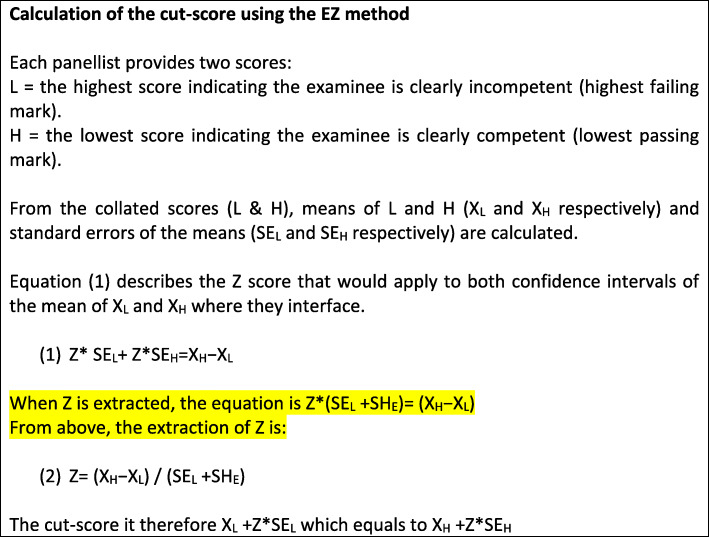
Calculation of the cut-score (EZ method)

The EZ method was introduced as a panellist-based method of which each member of a panel of experts in the topic covered by the examination, the curriculum and the expected learning outcomes, is requested to review all examination items and then provide numeric answers to the following two questions: (a) What would be the lowest score that indicates the examinee is *without any doubt*, clearly competent in the topics assessed (lowest passing mark = H); and (b) What would be the highest score that indicates the examinee is *without any doubt*, clearly incompetent in the topics assessed (highest failing mark = L). Then the means and standard errors of the means are calculated for the lowest passing mark and highest failing mark (Fig. 1).

Using these products, the calculated cut-score is the point where the distance between the determined cut-score and L (measured by z-score from data related to ‘L’) is equal to the distance between the determined cut-score and the H (measured by z-score from data related to ‘H’) (Fig. 1), hence that name ‘Equal Z method. It is possible and very likely that in the 2 z-scores would have different absolute values, yet, cut-score is placed where *the confidence that the cut-score is not a clear fail equals to the confident that the cut-score is not clear pass* .

When introduced, the EZ method was demonstrated with a panel of 17 panellists testing 20 multiple choice items. Although the results were promising, no comparison with other standard setting method was presented and the test items were used only for demonstration. The main objective of the current study was to assess the feasibility of the EZ method in real life circumstances, to assess its reliability and validity including undertaking a comparison of the results to another well-established standard setting method (Borderline Regression Method; henceforth BRM) [18].

## Methods

### Setting and sample

This study was conducted in a medical centre in north Taiwan. Medical students in Taiwan undertake OSCE in their final year, and the medical centre holds two sessions of mock OSCE every year prior students taking the national OSCE in April and May. Last-year medical students in the medical centre between June 2018 and May 2019 were recruited to this study. Noteworthy that medical training in Taiwan was changed from 7 years to 6 years in 2013, therefore this study used data of two cohorts of students in their last year in the program: 6th and 7th year during this period. The first mock OSCE was conducted in September and October 2018, and the second one was in January and February 2019.

The OSCE stations include topics of medicine, surgery, paediatric, obstetrics and gynaecology, and procedures. The tested skills include procedure, history taking, patient communication and education, and recognition of patient condition and treatment explanation.

Each mock OSCE includes two versions (round 1 and round 2) and students underwent only one of these versions by a simple randomisation. In each cohort about half of the students were examines in round 1 and the other half in round 2. Therefore, each student takes one round in the first mock OSCE and another round in the second mock OSCE. It is noted that the mock OSCE is a formative assessment and no overall pass or fail is provided to the students.

Regarding to determining the sample size, although there is no literature regarding to the number of examinees needed for borderline regression method, a conventional estimate with power 0.8 and alpha 0.05 suggested that the minimum examinees would be 20 [19]. Similarly, there is no literature suggesting the sample size for EZ model. However, our unpublished pilot study suggested that 99.99% of confidence could be obtained with 11 panellists.

### Marking sheets and standard settings

Traditionally this medical school uses borderline regression method to set the pass/fail cut-score. In the marking sheet, a set of items are listed to be marked as ‘completely achieved (two points)’, ‘partially achieved (one point)’ and ‘not demonstrated (zero point)’. The sum is added from all items in the station. Another mark is given as global ratings, and five ratings are as ‘bad (1)’, ‘need more efforts (2)’, ‘normal (3)’, ‘good (4)’ and ‘excellent (5)’. A linear regression then is calculated by global ratings as independent variable and sum of items as dependent variable. The cutting score is determined by sum of items, and it is calculated by setting global ratings as 2 (‘need more efforts’) in the regression formula within each station.

For the current study, 31 OSCE panellists volunteered to participate in the EZ standard setting method at the medical centre. Following a brief (10 min) introduction of how to set the two scores the panellists were given 50 min to review the first mock OSCE items (12 stations of round 1 and round 2) and individually indicating their highest passing mark and lowest passing mark for this OSCE (round 1). After a short break (10 min) the panellists were given another 50 min to repeat the process for the second mock OSCE (another 12 stations of round 1 and round 2).

### Data analysis

**Fig. 2:**
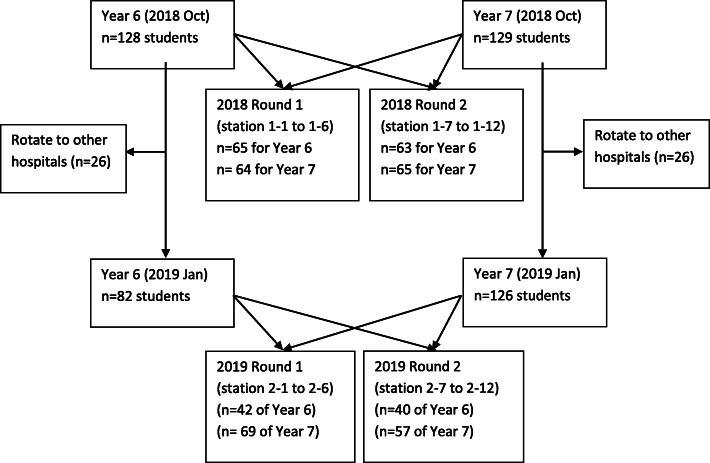
Flow chart of the OSCE

In this quantitative study, all data collected from mock OSCEs and EZ standard setting exercise were recorded to Microsoft Excel® for MS Office 365® electronically. Demographical data were reported in the flow chart (Fig. 2). For borderline regression, we calculated the slope, intercept, and standard error to generate the formula, cut point and the confidence interval for each station when global rating was set at 2. For the EZ method, mean and standard error for ‘lowest passing mark’ and ‘highest failing mark’ were calculated respectively; and the z-score were obtained to calculate the cut-scores. The z-scores were also used to calculate the levels of confidence. All cut scores and confidence intervals were presented in points out of 100 corresponding to the per-cent of correct items in the station.

Intraclass correlation measuring absolute agreement (henceforth ICC) was used to estimate the inter panel and inter method agreement, and paired t-test was performed to identify the differences among panels of EZ method and cohorts of examinees.

### Ethical review

The conduct of the study was approved by Institutional Review Board of the medical centre ref.: 2018–01-006CC.Consent was exempted for this minimal risk research.

## Results

A total of 257 and 208 medical students participated in 2018 and 2019 mock OSCE respectively (Fig. 2). The number of panellist for EZ method were 12, 10 and 9 respectively in three different days. Details of cut-scores in each station are shown in Tables 1 and 2 summarises the means of cut-scores for each standard setting methods and sessions. Overall, the results of this study demonstrate that the EZ method is reliable, yet it yields higher cut-scores than the BRM (Tables 1 and 2). The results also demonstrate consistency across groups within the BRM and across groups of panellists within the EZ Method (Tables 1 and 2).

**Table 1:** Cut-score yielded from the Borderline Regression and the EZ Methods

	Borderline Regression Method (year 6)			Borderline Regression Method (year 7)			EZ Method day 1		EZ Method day 2		EZ Method day 3		EZ Method overall	
	CS	95%CI		CS	95%CI		CS	Conf (%)	CS	Conf (%)	CS	Conf (%)	CS	Conf (%)
Station No.*		L	H		L	H								
1–1	49.20	46.52	51.87	40.81	38.04	43.59	49.47	100.00	58.98	99.95	55.71	93.20	54.87	100.00
1–2	41.88	39.02	44.73	51.51	49.35	53.66	60.63	99.75	57.62	99.67	59.29	95.45	59.28	100.00
1–3	47.70	44.24	51.17	49.04	46.78	51.30	62.11	98.80	56.63	99.62	59.15	92.86	59.60	99.99
1–4	53.96	50.83	57.10	63.12	60.36	65.88	74.37	97.18	70.57	95.41	75.32	94.75	73.53	99.87
1–5	64.87	62.03	67.71	64.89	62.71	67.08	65.97	99.50	61.39	98.56	71.05	99.23	66.21	99.99
1–6	58.70	55.89	61.51	59.70	56.66	62.73	51.71	99.26	54.34	96.81	52.15	92.72	52.84	99.97
1–7	42.32	39.79	44.85	42.42	39.62	45.22	56.07	99.87	51.61	99.74	59.71	96.34	55.64	100.00
1–8	51.00	48.23	53.77	49.09	44.23	53.96	58.11	99.84	53.27	98.04	62.62	90.30	57.91	99.97
1–9	66.55	63.70	69.39	65.03	63.28	66.77	60.01	99.92	57.13	99.51	59.46	95.29	58.67	100.00
1–10	35.56	33.08	38.03	29.31	26.77	31.86	53.70	99.61	54.27	93.70	59.53	88.08	55.82	99.89
1–11	63.18	60.83	65.54	62.35	59.03	65.67	62.40	98.97	63.18	96.91	66.74	86.67	64.04	99.90
1–12	52.82	49.11	56.54	50.77	47.94	53.59	62.83	97.65	63.59	95.62	68.22	93.13	64.69	99.89
2–1	55.55	53.71	57.38	49.67	47.28	52.06	55.51	99.57	61.53	99.88	58.70	96.66	58.10	100.00
2–2	41.19	39.24	43.13	43.52	42.09	44.94	52.07	99.98	52.01	99.24	54.18	96.66	52.74	100.00
2–3	55.07	52.72	57.43	55.97	53.99	57.96	59.11	97.59	52.78	98.08	64.31	95.79	58.63	99.93
2–4	66.55	63.46	69.63	54.71	52.23	57.19	69.75	94.04	66.99	97.41	72.39	85.13	69.61	99.43
2–5	61.61	58.61	64.60	61.71	59.73	63.68	64.94	99.07	64.37	97.08	68.79	85.24	66.00	99.91
2–6	50.93	46.67	55.19	49.17	47.35	51.00	57.35	98.73	51.07	98.76	58.42	95.34	55.75	99.98
2–7	57.32	55.38	59.26	49.81	48.07	51.54	61.41	98.60	59.27	99.94	61.93	98.49	60.92	100.00
2–8	44.33	42.56	46.10	46.44	44.10	48.79	59.56	99.57	59.22	98.89	61.71	93.16	60.10	99.99
2–9	51.32	48.31	54.33	57.68	54.15	61.21	57.96	99.03	55.83	99.45	58.24	97.12	57.42	100.00
2–10	56.17	52.46	59.88	38.61	34.15	43.06	60.47	95.94	56.46	98.22	62.03	95.00	59.73	99.92
2–11	64.82	59.95	69.68	66.33	63.37	69.29	68.72	97.48	70.63	94.95	72.61	91.67	70.53	99.85
2–12	55.96	51.45	60.48	59.65	56.32	62.98	71.12	93.27	70.51	97.65	76.44	98.17	72.35	99.83

*Stations 1–1 to 1–12 were of first mock OSCE, stations 2–1 to 2–12 were of second mock OSCE. First six stations were round 1 and latter six ones were round 2 in each mock OSCE

**Table 2:** Mean Cut-scores by method by session

Method	Exam/Session	CS	95%CI	
			Lo	Hi
BRM	BRM.Y6.CS	53.75	50.08	57.42
	BMR.Y7.CS	52.63	48.60	56.65
EZ	EZ.Day1.CS	60.63	57.98	63.27
	EZ.Day2.CS	59.33	56.72	61.95
	EZ.Day3.CS	63.25	60.44	66.06
	EZ Altogether	61.17	58.62	63.71

**Fig. 3:**
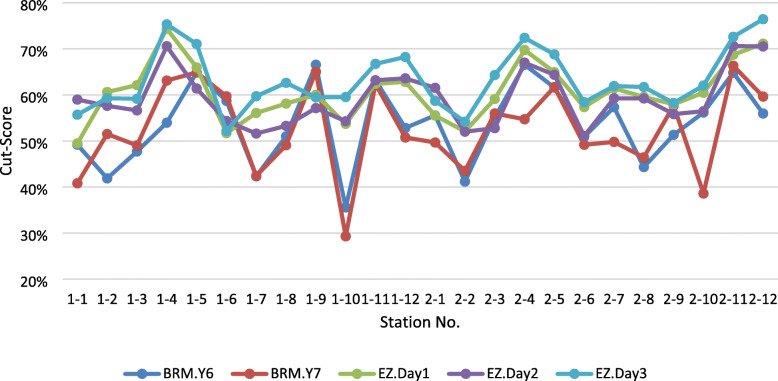
cut-scores by methods, sessions and stations

Intraclass correlation (ICC) measuring absolute agreement, across the three groups of panellists was .893 and .937 for the first and second rounds respectively (Table 3), demonstrating high level of agreement across groups with the EZ method. Within the BRM the ICC yielded similar values .938 and .744 for the first and second rounds respectively. Of note are the measures of confidence. The alignment between the BRM and the EZ method is also visually observed (Fig. 3).

**Table 3:** Intraclass correlation between EZ method and BRM cutscores

	BRM.Y6.CS	BMR.Y7.CS
EZ.Day1.CS	0.518	0.548
EZ.Day2.CS	0.56	5.46
EZ.Day3.CS	0.431	0.414

*p* < 0.01

The RBM yielded 95% CI ranged between 2.85 to 9.37 (mean = 5.60) points out of 100), whereas the EZ method used a direct method of confidence which ranged between 85 to 100% confidence (mean 96.71%) that the cut-score is neither clear pass nor clear fail. When the results from the 31 panellists were put together the confidence level was ≥99.43%.

The paired t-test results identified smaller differences between the cut-scores within methods than across methods (Table 4). Differences among cohorts using borderline regression method was not significant, while significant differences were identified among EZ method panels.

**Table 4:** Comparison of mean cut-scores across methods and sessions

		Mean	SD	SE	95% CI of the difference		t	df	*p*-value
					Lo	Hi			
First mock OSCE	BRM.Y6.CS - BRM.Y7.CS	0.08	5.30	1.530	−3.28	3.45	0.054	11	0.958
	EZ.Day1.CS - EZ.Day2.CS	1.17	4.34	1.254	−1.59	3.93	0.930	11	0.372
	EZ.Day1.CS - EZ.Day3.CS	− 2.67	3.45	0.995	−4.86	−0.48	−2.680	11	**0.021**
	EZ.Day2.CS - EZ.Day3.CS	− 3.83	4.20	1.211	−6.50	−1.17	−3.165	11	**0.009**
	BRM.Y6.CS - EZ.Day1.CS	− 7.33	9.91	2.861	− 13.63	−1.04	− 2.563	11	**0.026**
	BRM.Y6.CS - EZ.Day2.CS	− 6.17	9.34	2.696	−12.10	− 0.23	−2.287	11	**0.043**
	BRM.Y6.CS - EZ.Day3.CS	− 10.00	10.15	2.931	−16.45	− 3.55	−3.412	11	**0.006**
	BRM.Y7.CS - EZ.Day1.CS	− 7.42	9.10	2.627	− 13.20	−1.63	− 2.823	11	**0.017**
	BRM.Y7.CS - EZ.Day2.CS	− 6.25	9.75	2.815	− 12.45	−0.05	− 2.220	11	**0.048**
	BRM.Y7.CS - EZ.Day3.CS	− 10.08	10.57	3.051	−16.80	− 3.37	−3.305	11	**0.007**
Second mock OSCE	BRM.Y6.CS - BRM.Y7.CS	2.17	7.04	2.033	−2.31	6.64	1.066	11	0.309
	EZ.Day1.CS - EZ.Day2.CS	1.42	3.34	0.965	−0.71	3.54	1.468	11	0.170
	EZ.Day1.CS - EZ.Day3.CS	− 2.58	1.62	0.468	−3.61	−1.55	−5.519	11	**< 0.0001**
	EZ.Day2.CS - EZ.Day3.CS	− 4.00	3.41	0.985	−6.17	−1.83	−4.062	11	**0.002**
	BRM.Y6.CS - EZ.Day1.CS	− 6.42	5.00	1.443	−9.59	− 3.24	−4.446	11	**0.001**
	BRM.Y6.CS - EZ.Day2.CS	− 5.00	5.88	1.697	−8.73	− 1.27	−2.947	11	**0.013**
	BRM.Y6.CS - EZ.Day3.CS	− 9.00	5.29	1.528	− 12.36	− 5.64	−5.892	11	**< 0.0001**
	BRM.Y7.CS - EZ.Day1.CS	− 8.58	6.11	1.764	− 12.47	−4.70	− 4.865	11	**< 0.0001**
	BRM.Y7.CS - EZ.Day2.CS	− 7.17	6.34	1.829	− 11.19	− 3.14	−3.918	11	**0.002**
	BRM.Y7.CS - EZ.Day3.CS	− 11.17	6.04	1.744	−15.01	−7.33	− 6.401	11	**< 0.0001**

## Discussion

The main objective of this study was to assess the feasibility reliability and validity of the EZ method [17]. The discussion below focuses on each of these key features, which are most important when the quality of a standard setting is assessed.

### Feasibility

Undertaking the EZ method is relatively an easy process. The panellists need to review the whole examination items and determine only two marks: the highest score indicating the examinee is clearly incompetent (the highest failing mark) and the lowest score indicating the examinee is clearly competent (the lowest passing mark). In the conduct of this study each panel completed this task for an OSCE exam comprising 12 stations within less than 60 min. This time is comparable to the time required from panellists applying the Hofstee’s method [6] (p 209–215) and is much shorter than the time required for the Angoff or Bookmark methods [20, 21]. The current study employed 31 panellists whom all are busy clinicians, yet none had indicated that the task was too difficult or too time-consuming.

The main differences between the EZ method [17] and the Hofstee method [4] is related to the questions the panellists are required to address. Hofstee method [4] asked the panellists four questions: (1) What is the highest percent correct cut score that would be acceptable, even if every examinee attains that score? (2) What is the lowest per-cent correct cut score that would be acceptable, even if no examinee attains that score? (3) What is the maximum acceptable failure rate? and (4) What is the minimum acceptable failure rate? The EZ method [17] asks the panellists two questions only: (1 What would be the lowest score that indicates the examinee is without any doubt, clearly competent in the topics assessed? and (2) What would be the highest score that indicates the examinee is without any doubt, clearly incompetent in the topics assessed? The first two questions of Hosftee’s method [4] are quite similar to the two EZ method [17] question. Nonetheless, these questions are phrased in very different way. Hofstee’ questions focus on *acceptability* whereas the EZ method focuses on the panellist’s *confidence* without doubt that the mark indicated clear fail or clear pass. In other words, the questions asked by EZ method push the panellists to focus on certainty whereas Hosftee’s method pushes the panellists to focus on what they believe is acceptable by others. Trying to ascertain what others believe is acceptable adds more factors to the decision making and may involve some unintended social desirability biases [22]. On the other hand, unlike the Hofstee method, the EZ method has no consideration of the expected passing rate, and in that regard the EZ method is regarded as a criterion-based rather than a norm-based standard setting method.

The EZ method should also be compared to the Angoff method. The Angoff method employs panellists that review each examination item independently and then make a decision upon the probability that a hypothetical minimally competent examinee would answer that item correctly [2, 3] (p81–95). The Angoff method which is widely used is a very lengthy process compared to the EZ method and conceptually is far more challenging. The Angoff method asks panellists to make decision upon a hypothetical minimally competent examinee, which is pretty vague and subjected to individual perception of who is that minimally competent examinee. Then the panelist is required to estimate the probability that the hypothetical examinee would correctly answer each item. Estimating probabilities with no numeric data available has limited validity [23]. In comparison the EZ method asks a very simple and concrete question about what the panellist is certain about (minimum passing score and maximum failing score). In that regard, the EZ method seems to be superior to the Angoff method. On the other hand, it may be argued that estimating each item individually may be a more thorough process compared to the holistic approach utilised by the EZ and the Hofstee methods.

An important feature of feasibility is related to the statistical / technical skills required for calculating the cut-scores. The EZ method requires basic technical/statistical skills since the equations and formulae are readily available (Fig. 1) and everyone with basic mathematical skills would be able to calculate the cut-score using a simple calculator or a spreadsheet. This simplicity is shared with the Hofstee and the Angoff methods. On the other hand, other methods such as the Bookmark or the BRM [7] require advanced knowledge in statistics and psychometrics.

Overall, in terms of feasibility the EZ method requires relatively little time (about 60 min) to be implemented; about 10 panelists would yield sufficiently reliable results, it uses a simple language with clear criteria for judgment; and it requires basic mathematical skills with no need to access any advanced statistical psychometrical software. All of that makes the EZ method a feasible standard setting method.

### Reliability

Measuring the reliability of the EZ method is not a straight forward process. The EZ method does not require any particular level of inter-rater agreement since the disagreement is inherently presented in the confidence yielded from the standard error of the means of the lowest passing marks and the highest failing marks and is directly influencing the yielded cut-score. Thus, the appropriate measure of reliability is measuring *inter-panel agreement*. Yielding ICC value of .893 and .937 for the first and the second rounds, respectively, demonstrates very high level of inter-panel reliability. The other measure of reliability is the confidence that the cut-score is neither equal or higher than the lowest passing mark, nor equal or lower than the highest failing mark. This measure is calculated from the standard errors of the mean of the lowest passing mark and from mean of the highest failing mark as determined by the panellists (Fig. 1). With very few exceptions, the confidence levels were over 95% which is desirable. However, when the 31 panellists were considered as a single panel, the level of confidence was very high (≥99.43%). Note that it is possible to combine all panellists into one panel since each panellist makes the judgment independent to others with no communication between panellists during the process. Presenting a cut-score with the statistical confidence that it is neither equal or higher than the lowest passing mark nor equal or lower than the highest failing mark is very important for all stakeholders and obviously strengthens the defensibility of the cut-scores provided.

### Validity

An essential yet not sufficient evidence for validity is evidence for reliability, which has already been established above. On top of that, it is important to add that the EZ method has a slight advantage over most other methods by including the standard error of the means (SE) as an integral part of the cut-score calculation. This inclusion coupled with the measure of confidence provides important information on the determined cut-score. The yielded cut-score is a product of both panellists’ judgment and their level of agreement. No other standard setting methods has that unique and so informative feature.

Since there is no gold standard for any cut-score nor for any standard setting method [3, 24], a comparison of cut-scores produced from different standard setting methods cannot determine either validity or otherwise. Almost all previous studies that compared cut-scores yielded from different methods found significant differences in the cut-scores across method. The results of the current study are in line with that literature (Table 4). Nonetheless, despite the significant differences in the cut-scores between the EZ and the BRM methods, the level of agreement measured by Intraclass correlation (ICC) was acceptable (.414 < r < .556) (Table 2). The visual presentation of the agreement between methods also strengthens that argument (Fig. 3).

Furthermore, the EZ method offers some unique features, not explicitly addressed in other standard setting methods. The main point of difference is the focus on the thresholds between the clear pass or clear fail and the ‘borderline zone’ [25]. Although that focus had already been introduce by Willem Hofstee about four decades ago [4], the EZ method advances that approach by estimating and utilising the confidence around these two thresholds (‘lowest passing mark’ and ‘highest failing mark’). This utilisation leads to a new concept that emphasising the need to place the cut-score not necessarily at the nominal middle point between the lowest passing mark and highest failing mark, but rather at the point *where there is an equal chance for the cut score being neither lowest passing mark (or higher) nor being highest failing mark (or lower)* [17]. This feature is a major strength of the EZ methods since otherwise the cut-score might be biased either toward the passing mark or towards the failing mark [17].

### Limitations

This study may have some limitations. The most obvious one is that the comparison of the EZ method was made against only one other method (the BRM). Nonetheless, comparison of standard setting methods rarely involve more than two methods [26] and most comparisons were made between two methods only or within the same method under slightly different conditions [27–30]. Moreover, it has also been suggested that the variability of cut-scores within methods is as large as the variability across methods [31]. The current study demonstrates that the variability across methods was much greater than the variability within the methods (Table 2). Therefore, it is suggested that although more comparisons are recommended, the setting of this study was sufficiently robust. Another important limitation is that the comparison was made between methods that conceptually and technically are very different, which raises the question of what is really being compared? The BRM is a post-test, examination-based method, which requires no panellists, whereas the EZ method uses panellists only and is undertaken independently to the examination results. An alternative design might have been a comparison of the EZ method to the Angoff of the Hofstee methods, which would then provide more information on the particular features of the decision-making process across methods. Although desirable, such a design required resources that were not available for the current study. It is therefore strongly recommended that future studies would follow such a practice. The other limitation is that the optimal number of panellists for EZ method is not known yet, while a simulation study of Angoff method suggested 15 judges for precise estimates [3]. The comparison for the number of panellists could be critical for the time and cost for standard setting recruitment. Nonetheless, it must be noted that employing 15 panellist for a task of an hour would end up with 15 ‘person hours’ for 12 station OSCE; whereas Angoff and similar methods require the panellist to participate in a much lengthier process which would end up with many more ‘person hours’ [32].

## Conclusions

Overall this study suggests that the EZ method is a feasible, reliable and valid standard setting method. In summary it requires relatively little resources (takes about an hour to assess a 12 station OSCE); the calculation of the cut-score is simple and requires basic statistical skills; it is highly reliable even when only 10 panellists participate in the process; its validity is supported by comparison to another (very different) standard setting method (BRM), and it is statistically robust. All of that makes the EZ method worth the name we have given it ‘The EZ (easy) Method’.

## Data Availability

The datasets used and/or analyses of the current study are available from the corresponding author on reasonable request.

## Abbreviations

EZ method: Equal Z-score method
BRM: Borderline Regression Method
OBM: Objective Borderline Methods
OSCE: Objective Subjective Clinical Examinations
